# A Functional +61G/A Polymorphism in Epidermal Growth Factor Is Associated with Glioma Risk among Asians

**DOI:** 10.1371/journal.pone.0041470

**Published:** 2012-07-19

**Authors:** Xin Xu, Lei Xi, Jie Zeng, Qinhong Yao

**Affiliations:** Department of Oncology, The Affiliated Jiangyin Hospital of Southeast University Medical College, Wuxi, People's Republic of China; IPO, Inst Port Oncology, Portugal

## Abstract

**Background:**

Epidermal growth factor (EGF), a potent mitogenic protein, plays an important role in the development of cancers, including glioma. Previous studies showed that the EGF +61G/A polymorphism (rs4444903) may lead to an alteration in EGF production and/or activity, which can result in individual susceptibility to glioma. However, published data regarding the association between the +61G/A polymorphism and glioma risk was contradictory.

**Objective:**

The aim of this study was to perform a meta-analysis of eligible studies to derive precise estimation of the association of EGF +61G/A with glioma risk.

**Methods:**

We performed a pooled analysis of seven published studies that included 1,613 glioma cases and 2,267 controls. Odds ratios (ORs) and 95% confidence intervals (CIs) were used to assess the strength of the association. The pooled ORs were performed for codominant model, dominant model, and recessive model, respectively.

**Results:**

Overall, no significant associations between the EGF +61G/A polymorphism and glioma cancer risk were found for AA versus GG (OR = 0.95, 95% CI = 0.62–1.45), GA versus GG (OR = 0.94, 95% CI = 0.72–1.22), AA/GA versus GG (OR = 0.93, 95% CI = 0.70–1.23), and AA versus GA/GG (OR = 1.04, 95% CI = 0.77–1.39). However, in the stratified analysis by ethnicity, the EGF +61G/A polymorphism had a higher risk of glioma development among Asians, but a lower risk among Caucasians.

**Conclusions:**

Taken together, the results suggest that the EGF +61G/A polymorphism may contribute to the susceptibility of glioma in different ethnic groups.

## Introduction

Gliomas are the most common type of primary brain tumors, which accounts for more than 40% of the newly diagnosed brain tumors [1]. According to the clinicopathological features, gliomas can be divided into astrocytic tumors, oligodendrogliomas, and oligoastrocytomas [2]. The etiology of glioma is largely unknown. High-dose ionizing radiation is known to increase risk [3]. However, this is likely to account for only a small proportion of cases. Furthermore, only a small proportion of exposed individuals will develop gliomas, suggesting a genetic predisposition of gliomas.

The epidermal growth factor (EGF) gene is located in chromosome 4q25-27. It is a member of the EGF superfamily, which includes transforming growth factor-α, heparin-binding EGF-like growth factor, epiregulin, betacellulin, and amphiregulin [4]. As a growth factor, EGF can activate DNA synthesis and cellular proliferation and stimulate mitogenesis in epidermal tissue [5]. Epidermal growth factor receptor (EGFR), an important receptor of EGF with high affinity, was often overexpressed in glioma cells [6]. The EGFR gene is frequently amplified and overexpressed in human glioblastomas [7]. Previously, Shahbazi et al. identified a G to A polymorphism at position 61 (+61G/A, rs4444903) in the 5′-untranslated region of EGF, and the presence of the variant A allele led to a decreased EGF production in peripheral blood mononuclear cells cultures, glioblastoms, and gliomas cell lines [8], [9].

To date, a number of studies have reported the association between the EGF +61G/A polymorphism and glioma risk, but the results were inconclusive. Two published articles tried to find this association by performing meta-analysis [10], [11]. However, these two studies had the same limitation that the sample size was relatively small. Hence, a meta-analysis of seven case-control studies involving 1,613 cases and 2,267 was performed to derive a more precise estimation of the association of EGF +61G/A with glioma risk.

## Materials and Methods

### Publication search

One online electronic database (PubMed) was searched using the search terms ‘epidermal growth factor’ or ‘EGF’, ‘polymorphism’, and ‘glioma’, ‘genetic susceptibility’, single nucleotide polymorphisms, ‘germline variation’ (from January 1, 2000 to August 1, 2011). The search was limited to English-language papers. Additional studies were identified by a hand search of the references of original studies. Of the studies with the same or overlapping data published by the same investigators, we selected the most recent ones with the largest number of subjects.

### Inclusion and exclusion criteria

The inclusion criteria were (a) on EGF +61G/A polymorphism and glioma risk, (b) case-control studies, and (c) containing useful genotype frequencies. Major exclusion criteria were (a) no control population, (b) no available genotype frequency, and (c) duplication of the previous publication.

### Data extraction

Information was carefully extracted from all eligible publications independently by two investigators according to the inclusion criteria listed above. When it came to conflicting evaluations, an agreement was reached after a discussion. For each study, the following data were considered: the first author's last name, year of publication, country of origin, ethnicity, exposure to high-dose ionizing radiation, and numbers of genotyped cases and controls. Different ethnic descents were categorized as Caucasian and Asian. We did not define any minimum number of patients to include a study in our meta-analysis.

### Statistical analysis

The strength of association between the EGF +61G/A polymorphism and glioma risk was assessed by calculating crude odds ratios (ORs) with 95% confidence intervals (CIs). The pooled ORs were performed for codominant model (AA versus GG; GA versus GG), dominant model (AA/GA versus GG), and recessive model (AA versus GA/GG), respectively. Stratified analyses were also performed by ethnicity and sources of controls. In consideration of the possibility of heterogeneity across the studies, a statistical test for heterogeneity was performed based on the Q statistic [12]. If the *P*>0.05 of the Q-test which indicates a lack of heterogeneity among studies, the summary OR estimate of each study was calculated by the fixed-effects model (the Mantel-Haenszel method) [13]. Otherwise, the random-effects model (the DerSimonian and Laird method) was used [14]. The stability of the meta-analysis' results was accessed by performing one-way sensitivity analyses [15]. The potential publication bias was estimated using Egger's linear regression test by visual inspection of the Funnel plot. If *P*<0.05, statistically significant publication bias existed [16]. All statistical tests were performed with the software STATA version 10.0 (Stata Corporation, College station, TX, USA).

## Results

### Characteristics of Studies

A total of seven eligible studies involving 1,613 cases and 2,267 controls were included in the pooled analyses [9], [17], [18], [19], [20], [21]. The characteristics of selected studies are summarized in Table 1. There were four studies of Caucasian descendants and three studies of Asian descendants. Gliomas were confirmed histologically or pathologically in most studies [9], [17], [19], [20], [22]. A classic polymerase chain reaction-restriction fragment length polymorphism assay was performed in four studies [17], [19], [20], [22]. Other three studies were using TaqMan [9], [21] or PCR-LDR [18] methods. In addition, several studies were excluded because of lacking genotyping information or control subjects. The distribution of genotypes in the controls of each study was in agreement with Hardy-Weinberg equilibrium except for one study [21].

**Table 1:** Characteristics of studies included in the meta-analysis.

Author (year)	Ethnicity (country)	Source of controls	Sample size (case/control)	Genotype frequencies (GG/GA/AA)		Hardy-Weinberg equilibrium (controls)
				case	control	
Bao (2011)	Asian (China)	Hospital	160/320	0.400/0.444/0.156	0.441/0.472/0.088	0.162
Wang (2010)	Asian (China)	Population	672/693	0.443/0.454/0.103	0.528/0.398/0.074	0.917
Pinto (2009)	Caucasian (Brazil)	Hospital	165/200	0.285/0.479/0.236	0.280/0.460/0.260	0.260
Liu (2009)	Asian (China)	Hospital	168/194	0.446/0.464/0.089	0.474/0.443/0.082	0.510
Costa (2007)	Caucasian (Portugal)	Hospital	197/570	0.284/0.492/0.223	0.230/0.467/0.304	0.142
Vauleon (2007)	Caucasian (France)	Population	209/214	0.211/0.488/0.301	0.145/0.561/0.294	0.031
Bhowmick (2004)	Caucasian (USA)	Hospital	42/76	0.357/0.381/0.262	0.158/0.487/0.355	0.909

### Quantitative synthesis

**Figure 1:**
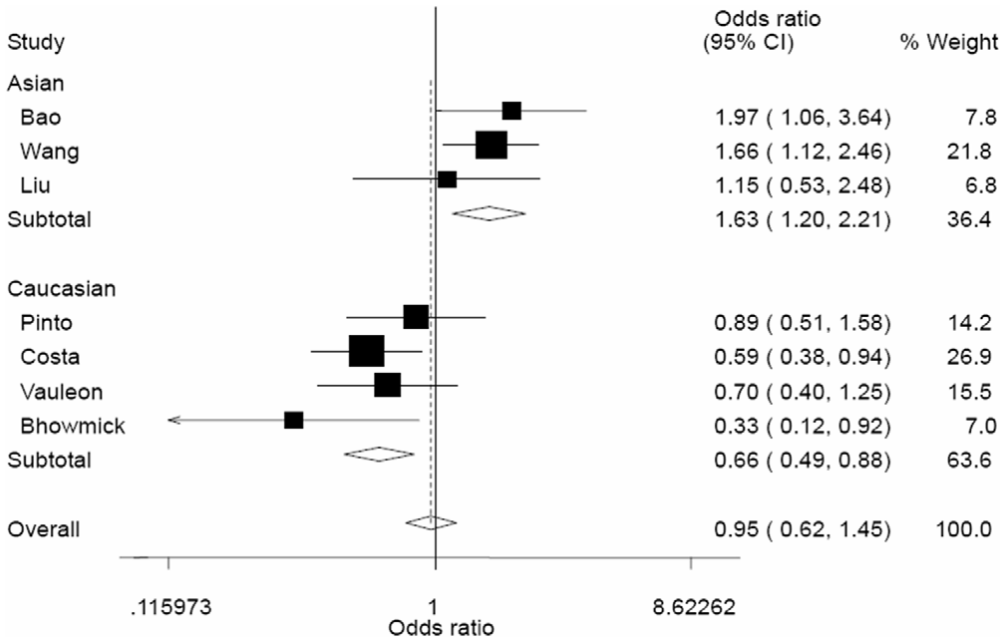
Begg's funnel plot for publication bias test (AA vs. GG). Each point represents a separate study for the indicated association. Log[or], natural logarithm of OR. Horizontal line, mean effect size.

**Figure 2:**
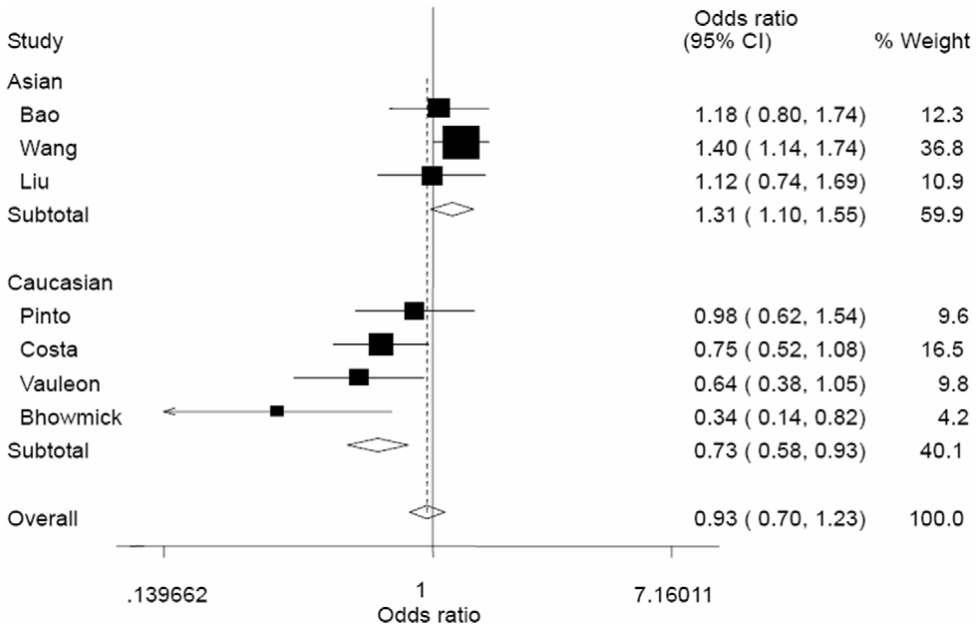
Begg's funnel plot for publication bias test (AA/GA vs. GG). Each point represents a separate study for the indicated association. Log[or], natural logarithm of OR. Horizontal line, mean effect size.

As shown in Table 2, no significant associations between the EGF +61G/A polymorphism and glioma risk were observed in all genetic models. Overall, no significant associations were found for AA versus GG (OR = 0.95, 95% CI = 0.62–1.45), GA versus GG (OR = 0.94, 95% CI = 0.72–1.22), AA/GA versus GG (OR = 0.93, 95% CI = 0.70–1.23), and AA versus GA/GG (OR = 1.04, 95% CI = 0.77–1.39). However, in the stratified analysis by ethnicity, significantly increased risk of glioma was found among Asians in all genetic model (AA versus GG, OR = 1.63, 95% CI = 1.20–2.21; GA versus GG, OR = 1.25, 95% CI = 1.04–1.49; AA/GA versus GG, OR = 1.31, 95% CI = 1.10–1.55; AA versus GA/GG, OR = 1.48, 95% CI = 1.11–1.98). In contrast, the EGF +61G/A polymorphism was associated with a significant decreased risk of glioma in homozygote comparison (AA versus GG, OR = 0.66, 95% CI = 0.49–0.88, Figure 1) and dominant model (AA/GA versus GG, OR = 0.73, 95% CI = 0.58–0.93, Figure 2). In the stratified analysis by sources of controls, no significant results were observed in all genetic models.

**Table 2:** Meta-analysis of the EGF +61G/A polymorphism on glioma.

Variables	n^a^	AA vs. GG		GA vs. GG		AA/GA vs. GG (dominant)		AA vs. GA/GG (recessive)	
		OR (95% CI)	*P* b	OR (95% CI)	*P* b	OR (95% CI)	*P* b	OR (95% CI)	*P* b
Total	7	0.95 (0.62–1.45)^c^	0.001	0.94 (0.72–1.22)^c^	0.016	0.93 (0.70–1.23)^c^	0.002	1.04 (0.77–1.39)^c^	0.024
Ethnicities									
Asian	3	1.63 (1.20–2.21)	0.560	1.25 (1.04–1.49)	0.447	1.31 (1.10–1.55)	0.537	1.48 (1.11–1.98)	0.473
Caucasian	4	0.66 (0.49–0.88)	0.372	0.77 (0.60–1.00)	0.165	0.73 (0.58–0.93)	0.187	0.81 (0.64–1.02)	0.415
Source of controls									
Population-based	2	1.11 (0.48–2.57)^c^	0.016	1.20 (0.98–1.47)^c^	0.005	0.98 (0.45–2.12)^c^	0.005	1.24 (0.94–1.64)	0.249
Hospital-based	5	0.87 (0.51–1.48)^c^	0.009	0.94 (0.77–1.16)	0.256	0.93 (0.77–1.13)	0.070	0.95 (0.63–1.42)	0.037

a Number of comparisons.

b *P* value of Q-test for heterogeneity test.

c Random-effects mode.

### Test of heterogeneity, sensitivity analyses and publication bias

There were significant heterogeneity between studies in overall comparisons (Table 2). A single study involved in the meta-analysis was deleted each time to reflect the influence of the individual data-set to the pooled ORs, and the corresponding pooled ORs were not materially altered (data not shown). Begg's funnel plot and Egger's test were performed to assess the publication bias of literatures. The shape of the funnel plots seemed asymmetrical in dominant model comparison, suggesting the presence of publication bias (figures not shown). Then, the Egger's test was adopted to provide statistical evidence of funnel plot asymmetry. As expected, the results shown an obvious evidence of publication bias (t = −3.83, *P* = 0.012 for AA/GA versus GG).

## Discussion

This meta-analysis examined all the available data on the association between the EGF +61G/A polymorphism and glioma risk, including a total of 1,613 glioma cases and 2,267 controls. We found that the variant genotypes of the +61G/A polymorphism were associated with significant increased risk of glioma among Asians, and decreased risk among Caucasians. Given the important roles of EGF in cell proliferation, differentiation or survival through binding to EGFR, it is biologically plausible that EGF polymorphism may modulate the risk of glioma.

Genetic variants occurred in EGF promoter region may influence gene expression, dysregulate EGF signaling, and lead to neoplastic transformation [23], [24]. The EGF +61G/A polymorphism was well characterized in both functional analyses and association studies. It has been shown that the +61G/A substitution led to a decreased EGF production *in vitro* and decreased risk of malignant melanoma [8]. To date, no phonotypical studies were reported to evaluate the G-1380A and A-1744G variants. They may alter plasma EGF levels together with +61G/A and result in disturbance of the EGF signaling. Bhowmick et al. found that the +61G/A polymorphism was associated with risk in aggressiveness of glioblastoma [9]. However, three studies showed that there was no association between the +61G/A polymorphism and glioma risk [19], [20], [21]. The results of our meta-analysis were consistent with these experimental findings. Nevertheless, the +61G/A polymorphism was not specially associated with glioma risk. A meta-analysis with 21 case-control studies containing 5,768 cases and 8,841 controls has investigated the relationship between the EGF +61G/A polymorphism and risk of many types of cancers, the results found that the +61G/A polymorphism was associated with the risk of gastric cancer, esophageal cancer, hepatoma and glioma, but not melanoma and breast cancer [10].

Our results showed that the EGF +61G/A polymorphism had a significant increased risk of glioma in Asians, but decreased risk in Caucasians, suggesting a possible role of ethnic differences in genetic backgrounds and the environment they live in [25]. The influence of the 61A allele might be masked by the presence of other as-yet unidentified causal genes involved in glioma development in Caucasians. In addition, it was also likely that the observed ethnic differences may be due to chance because studies with small sample size may have insufficient statistical power to detect a slight effect or may have generated a fluctuated risk estimate [26]. Other factors such as selection bias, different matching criteria may also play a role. The above differences may account for the inconsistent results.

Recently, in order to identify common, low-penetrance cancer susceptibility genes, large genome-wide association studies (GWAS) have been performed, including glioma. GWAS of glioma in populations of European ancestry were completed in the United States and the United Kingdom [27], [28]. However, the biological pictures being revealed by GWAS are still largely incomplete. Many of the associations identified by GWAS do not involve previous candidate genes for a particular disease, and many associated markers are in genomic locations in unknown gene regions [29]. Therefore, it is predicted that the pace of discovery will further as a result of second-generation GWAS, following on analyses and meta-analyses.

Some limitations of this meta-analysis should be addressed. First, the eligible studies were only published studies included in the meta-analysis; therefore, publication bias have occurred in our study. Second, the subgroup used for meta-analysis for interactions between EGF polymorphism and cigarette smoking did not include all eligible studies, because it was not possible to obtain information from some studies on smoking status. Third, meta-analysis remains retrospective research that is subject to methodological deficiencies. Despite these limitations, a detailed protocol initiating the analysis was included, by performing standardized methods for study selection, data extraction, and data analysis.

Our meta-analysis suggested that the EGF +61G/A polymorphism may be associated with an increased glioma risk among Asians, but a decreased glioma risk among Caucasians. However, in the stratification by source of controls, no significant association was found with respect to glioma risk in the EGF +61G/A polymorphism. Larger and well-designed multicentric studies based on the same ethnic group and other cancers are warranted to validate our findings.
